# Managing intra-EU mobility—do WHO principles of ethical recruitment have relevance?

**DOI:** 10.1186/s12960-017-0247-7

**Published:** 2017-11-09

**Authors:** Réka Kovács, Edmond Girasek, Eszter Kovács, Zoltán Aszalós, Edit Eke, Károly Ragány, Zoltán Cserháti, Miklós Szócska

**Affiliations:** Health Services Management Training Centre, Semmelweis University, Kútvölgyi út 2, Budapest, Hungary

**Keywords:** Human resources for health, Ethical recruitment, WHO Code, Circular migration, Health workforce planning, Mobility data

## Abstract

**Background:**

The WHO Global Code of Practice on the International Recruitment of Health Personnel provides for guidance in health workforce management and cooperation in the international context. This article aims to examine whether the principles of the voluntary WHO Global Code of Practice can be applied to trigger health policy decisions within the EU zone of free movement of persons.

**Methods:**

In the framework of the Joint Action on European Health Workforce Planning and Forecasting project (Grant Agreement: JA EUHWF 20122201 (see healthworkforce.eu)), focus group discussions were organised with over 30 experts representing ministries, universities and professional and international organisations. Ideas were collected about the applicability of the principles and with the aim to find EU law compatible, relevant solutions using a qualitative approach based on a standardised, semi-structured interview guide and pre-defined statements.

**Results:**

Based on implementation practices summarised, focus group experts concluded that positive effects of adhering to the Code can be identified and useful ideas—compatible with EU law—exist to manage intra-EU mobility. The most relevant areas for intervention include bilateral cooperations, better use of EU financial resources, improved retention and integration policies and better data flow and monitoring.

Improving retention is of key importance; however, ethical considerations should also apply within the EU. Compensation of source countries can be a solution to further elaborate on when developing EU financial mechanisms. Intra-EU circular mobility might be feasible and made more transparent if directed by tailor-made, institutional-level bilateral cooperations adjusted to different groups and profiles of health professionals. Integration policies should be improved as discrimination still exists when offering jobs despite the legal environment facilitating the recognition of professional qualifications. A system of feedback on registration/licencing data should be promoted providing for more evidence on intra-EU mobility and support its management.

**Conclusions:**

Workforce planning in EU Member States can be supported, and more equitable distribution of the workforce can be provided by building policy decisions on the principles of the WHO Code. Political commitment has to be strengthened in EU countries to adopt implementation solutions for intra-EU problems. Long-term benefits of respecting global principles of the Code should be better demonstrated in order to incentivise all parties to follow such long-term objectives.

## Background

The voluntary WHO Global Code of Practice on the International Recruitment of Health Personnel (hereinafter the Code) [1] was adopted by the 63rd World Health Assembly on 21 May 2010 to address the challenge of enhanced migration especially from lower income countries, with a focus on strengthening health systems. Two reporting cycles have passed, with significant improvements concerning quality and content and 37% increase in the number of designated national authorities (with major improvement in certain regions, e.g. Western Pacific) [2]. In 2015, the first review of the relevance and effectiveness of the Code—after examining evidence on how the Code informed and promoted policy dialogue—concluded that the Code remains “highly” relevant, particularly in the context of increasing intra- and inter-regional labour mobility. They found that many countries still have significant reliance on foreign-trained health personnel, and trends—e.g. population growth, ageing of population and health workforce, urbanisation, increasing liberalisation of rules related to skilled migration—continue to drive global supply and demand constraints. This makes the principles and provisions of the Code increasingly essential to health systems strengthening worldwide [3, 4].

Main directions and magnitude of health professional mobility within the European Union have recently been studied, and significant changes in mobility patterns as a consequence of the EU enlargements in 2004 and 2007 and the 2008 economic crises were identified, although the available data is still limited. Results show that outflows from 12 new EU Member States (EU-12) increased substantially after accession and later decreased but remained at a higher overall scale than before EU enlargement [5, 6]. The economic crises also had negative consequences on the availability of professionals, with a complex picture of movements still following the East-West, South-North pattern in main directions. While all Member States are losing, many EU-15 countries (old Member States) are simultaneously receiving health professionals [5, 6]. The effects of intra-EU mobility are complex; destination and source countries experience positive and negative effects on the efficiency and equity of EU health systems, such as balancing supply and demand, filling up shortage posts, sending home remittances, gaining expertise in a different system as merits, and reliance on unstable foreign workforce, integration difficulties, growing imbalances in availability, growing burden for home-remaining staff and decreased access to quality health care in regions affected by outflow as drawbacks. There is a risk however that mobility disproportionally benefits the wealthier at the expense of less advantaged EU Member States [7].

The latest data on international migration clearly show the growing ratio of intra-EU migrants in the biggest destination countries in the EU like Germany and the UK [8]. Changes in Member States international recruitment policies originate also from WHO Code implementation, resulting in reduced or even legally banned recruitment activity in countries on the WHO list of severe shortages. Despite the existence of Directive 2005/36/EC on the mutual recognition of professional qualifications ensuring automatic recognition in five health professions (doctors, dentists, pharmacists, nurses and midwives), there is evidence that professionals might perform tasks below their previous skill level when moving within the EU [6], which raise ethical questions concerning the integration of migrants. Movements towards better professional and financial possibilities—facilitated by the eased recognition of qualifications—may result in deepening inequalities in the sending regions, where remaining workforce has to face higher workload, and decreasing availability of workforce in shortage professions and underserved areas can even endanger the provision of services. The new emerging map of Europe’s mobility flows thus raise new ethical and policy questions concerning the scope of intra-EU solidarity [6].

While respecting the right to free movement, ensuring equal access to quality care is also the clear endeavour of EU health ministers expressed in Council Conclusions [9]. These objectives should be taken into account when establishing or modifying EU Member States’ recruitment and retention policies, and also, health workforce planning initiatives and interventions should consider the effects of the free movement of persons across borders. It is rather challenging, since limitations of available data on health professional mobility constitute a constraint for effective planning [5], making projections especially difficult in Member States with considerable workforce flows [10].

The question arises, how to successfully mitigate the negative effects of free movement on health care delivery in certain countries and regions within the EU. At EU level, the Action Plan on health workforce [11] embraces many areas where common challenges should be tackled and European cooperation and good practice sharing should be fostered. The key areas include improving health workforce planning and forecasting, anticipating future skills needs, improving recruitment and retention, while concerning international recruitment EU policies and actions supporting the implementation of the Code and reinforcing Member States’ commitment to it. In the ambit of this Action Plan—among others—a Joint Action programme was initiated in health workforce planning and forecasting, and also a study has been commissioned by the European Commission to support EU Member States retention policies with useful practices [12].

EU Member State level good practices aiming to implement certain principles of the Code are available in growing number including bilateral agreements fostering circular migration [13, 14]. While lacking a clear definition, elements of this phenomenon collected within the Joint Action include being non-permanent, involving at least two countries, more than one cycle and a situation which is beneficial for both the sending and the receiving countries and the migrant individual [14]. The definition by the International Organisation for Migration includes temporary and long-term circular migration, which is voluntary and beneficial if linked to labour market needs [15], while other authors distinguish between spontaneous/voluntary and managed forms [13]. The benefits of circular migration continue to be debated in the literature, stating also that bilateral agreements have less relevance; the largest labour movement between countries takes place outside these channels, through recruitment agencies, family links and social networks [7].

The aim of this article is to examine whether certain principles of the Code can be applied to trigger health policy decisions in countries within the EU free movement zone.

## Methods

The results presented in this paper are based on the workshop activities conducted as part of the Joint Action on European Health Workforce Planning and Forecasting project (JA EUHWF 20122201) and are presented in detail in the “Report on the applicability of the WHO Global Code of Practice on the International Recruitment of Health Personnel within a European context” of Work Package 4 (WP4) “Data for improved health workforce planning” [16].

Discussions were organised at two occasions (Bratislava, Lisbon) having a considerable overlap of participants from the Joint Action. The aim of the discussions was to explore how health workforce planning experts see the possibility of applying the principles of the Code as guidance to manage mobility within the free movement zone of the EU. Presentations of the implementation of the Code in relation to third countries and also recent and current EU measures and policies together with related activities of social partners (including EPSU-HOSPEEM Code of Conduct) have been introduced to provide inputs for the discussions. Based on a qualitative approach, a standardised, semi-structured interview guide supported the first workshop to collect ideas about EU law compatible, relevant solutions. Twelve core topics resulted from this exercise with thematic content analysis as issues of major interests, as presented in Table 1. The second discussion was organised in four focus groups including seven to eight persons/group, altogether more than 30 participants from European professional organisations, national ministries, universities and international organisations. Experts represented both source and destination countries. Pre-defined, provocative statements triggered the discussions, which had to be revised by consensus. In the end at a plenary, all participants voted on the relevance and feasibility of the statements in the EU. Participants could express their preferences by using three “+” marks for the most relevant/feasible action and one “0” mark for the least relevant/feasible one. All 31 participants voted with the three “+” marks (93 “+” votes together), but 13 persons were not willing to use the “0” mark, signalling that all topics were very relevant (18 “0” votes given). When evaluating the vote, top importance was given to statements of which the summation evaluation (“+” mark minus “0” mark) was above 10, high importance above 5, medium above 0 and low under 0.

**Table 1:** Core topics relating to the applicability of the Code and their relevance

Grouping	Core topic	Number of vote of relevance	Number of vote of lower relevance
Top	Retention policies	23	–
Top	Compensation	18	–
Top	Circular migration	14	–
High	Equal treatment of foreign health workforce	9	–
High	Data and information exchange	7	–
Medium	Awareness raising	5	–
Medium	An EU handbook on best practices	4	–
Medium	Individuals’ motivation as the most important factor	4	1
Medium	Cooperation in the field of graduate and postgraduate training	5	3
Medium	Regulating recruitment agencies’ activities	2	1
Low	Professional organisations’ involvement	2	3
Low	EU level Code of Conduct	–	10

## Results

Results showed that from 12 core topics identified as most relevant from the context of the Code to ensure sustainable EU health workforce, five issues can be identified as top and high priorities which are worth concentrating on first when applying the Code within the circumstances of free workforce mobility [16].

### Retention policies

Article 3.6 of the WHO Code encourages Member States to work towards retention strategies that will reduce their need to recruit migrant health personnel*.* Experts highlighted that awareness raising is needed as the Code is often narrowly interpreted as implying only international recruitment. They considered that as international recruitment cannot be banned, commitment to training and retaining has to be reinforced at all levels. Top importance should be given to retention policies also because mobility is facilitated by the legislative framework of the EU. As countries experiencing considerable inflows can also face problems of retaining their workforce—the example of Ireland shows it evidently [16]—introducing effective retention policies is in the interest of all Member States.

Experts argued that national-level retention practices have to be supplemented by cross-country/bilateral and European level solutions, even if EU level actions are difficult to suggest. Retention measures are aimed at the individual health worker, whose choice to stay can be fostered. The Commission’s collection of best practices [12] emphasises that a group of measures is to be introduced in order to influence individuals’ choices. Experts also listed actions, e.g. incentive working conditions, institutional level trainings for skills development, proper career progress, regional incentives in case of geographical maldistribution, bonuses for certain specialties and educational loans with a possibility of later remittal. Concerning the key factor of remuneration, experts concluded that competing with some Member States’ salary levels is not an option in many countries.

### Compensation mechanisms

In Article 5.2, the WHO Code calls for countries to provide among others technical assistance, support for retention and training of health professionals in countries they recruit from, although there is no explicit mention of financial compensation (only financial support to strengthen capacity in Article 10.3.). Training health professionals is a costly investment, as reinforced by the experts. Mechanisms for possible compensation are considered in some countries; however, examples of real investment remain unknown or are developing. Some EU countries are considering to, or have introduced systems, where the reimbursement of state-financed training costs are imposed on professionals if they do not work for a given period in their respective health systems [17]. Hungary for example from 2012 requires a period of work equal to studies in state-financed higher education after finishing training. As this period of work must be completed within 20 years after graduation, effects on retention can only be analysed at a later stage. The implementation of these types of solutions, however, raises various challenges.

Although experts acknowledged that ethical considerations also exist within the internal market, agreeing on an EU-level system of compensation raised some concerns. Even stating that there is some responsibility to strengthen training and retention in countries where a significant number of health professionals arrive from, it is considered difficult to find the basis for such reimbursement. Experts supported national solutions, including training fees, which nevertheless may cause difficulties in countries where training was traditionally free of charge. Experts also regarded exchange programmes for medical school teachers and knowledge-transfer as compensation to a certain extent. EU financial mechanisms could also be considered to see how they can be better used for the purpose of health workforce retention. More focus and commitment is needed primarily at national level when deciding on operational programmes.

### Circular migration—bilateral agreements

According to Article 3.8 of the WHO Code Member States should facilitate circular migration of health personnel, so that skills and knowledge can be extended to the benefit of both source and destination countries.

Experts found especially difficult to estimate the added value of bilateral agreements promoting circular mobility within the EU, where health workers can take up jobs freely in other Member States and their professional qualifications are recognised. Examples were provided by them on structured migration [13, 14, 16]. In the Irish example in the frame of the International Medical Graduate Training Initiative, a structured migration/training route is offered for Pakistani doctors whereby they can obtain 2-year postgraduate specialty training in Ireland, matched to their needs, which is accredited by their training college in Pakistan. These doctors also serve local needs in Ireland and must return to their source country after 2 years of training to be awarded qualification. In the German example—The Triple Win® Approach—foreign nurses are recruited and get language training in the country of origin. Support is provided with their transfer to Germany, including help with work permit, residence and qualification recognition. When working in Germany, they are further educated in geriatric and elderly care. They will be supported to utilise experience gained abroad in their home country; however, moving back home should be incentivised, e.g. by creating jobs [7]. Limited evidence exists on intra-EU solutions and the possible EU-level actions that could facilitate circular mobility, so further sharing of good practices is necessary. The urgent need for further data and research was reinforced alongside proposing the consideration of needs of the source country as the starting point of such solutions [14].

Experts found institutional-level cooperation the most feasible in the EU context. Graduate and postgraduate exchange programs can be executed, such as the medical university cooperation between Hungary and Sweden for the short-term exchange of pathologists. This exchange programme provides short-term (maximum 3 months) exchange for Hungarian pathology students and pathologists, who return after the completion of this period. This programme aims bilateral knowledge sharing and is also beneficial for all parties as it helps to cover short-term needs of the Swedish health system. Experts considered that circular mobility can also be planned into career pathways in a systematic way to serve individual career development purposes. Length depends on the situation; even very short-term exchanges can be justified by the needs of a health system. The needs and aspirations of different age groups and also various life situations have to be taken into account. National-level measures cannot be excluded either and can for example support institutional-level programs in order to provide a framework. As of ultimate importance, source countries need to close the circle by creating jobs and improving conditions in order to offer attractive possibilities for returning professionals [16].

### Equal treatment of foreign health personnel

Articles 4.4–4.6 of the WHO Code focus on the fair treatment of migrants stating that equal treatment has to be provided with domestically trained personnel in terms of employment and conditions of work, and also, they should be provided opportunities and incentives to strengthen professional education, qualifications and career progression on the basis of equal treatment.

Experts referred to the “brain waste” as working below the skill level that might cause professional dissatisfaction of the individual health professional, a waste of his/her skills and a waste for the entire health system of the receiving country as well. They also added that accepting a lower level job can only be justified if it is the choice of the individual based on clear information on job content. Language can also be a possible difficulty in the integration of EU migrants, as without proper language skills, professionals can easily find themselves in lower level jobs. As adequate level of language knowledge is of great importance from the point of view of patient safety, language training offered to arriving migrants supporting their integration is in the spirit of the WHO Code.

Better provision of information on the job contents supported by a legal framework ensuring transparency, better job classification together with salary and benefit packages and an agreement on the professional side on a language certificate/evaluation to enhance patient safety are among the ideas mentioned by experts as supporting integration. An agreement of the social partners, named the EPSU-HOSPEEM Code of Conduct [18] on the ethical recruitment of health professionals, could also have more significance when employing foreign workforce. In addition to mapping and monitoring Continuous Professional Development practices at the European level, continuous updating and recognition are also of relevance [16].

### Data and monitoring

The Code encourages improving data collection, using collected data for monitoring, analysis and policy formulation (Article 6) and establishing or strengthening information exchange at local and global level (Article 7). The discussion strengthened the argument that with rapidly changing mobility patterns, there is a need for accurate health workforce data. Experts agreed that international data collections have their limits, especially when addressing comparability [19]. Data collected at the international level can only be as good as the data provided by national bodies. Therefore, it is worth investing in health workforce data collection systems at national level [16, 19, 20].

Outflow data is of great importance in planning future health workforce. Reliable information on outflow is challenging since health workers are free to leave without any notification about their new jobs in another EU Member State. It is argued that data capturing on out-migration is only feasible if information on in-migration is shared [21]. Source countries often underline the need for cooperation with destination countries about data provision on mobility. To introduce an automatic information exchange based on existing structures within the EU was found feasible by experts. Recipient/target countries have data on new entrants; thus, the possibility exists for data exchange. Tracking individual movements does not seem feasible and is not a common practice with any other profession and would also raise data protection issues. Health professionals could most probably only be tracked for statistical reasons, as the level of influence on domestic stock might justify this approach. Language exams could also provide some additional information on mobility flows, especially with professions requiring more direct professional-patient contact [16].

## Discussion

Results highlighted the most feasible cooperation areas in mobility management within the European Union in the spirit of the Code.

While acknowledging the conclusions of the Commission study on the complexity of retention policies [12], experiences from recent years in Central and Eastern Europe show the decisive nature of the remuneration [17]. Country case studies show the direct relationship between salary increase and the intention to migrate (Poland) and identify salary and quality of life as top push factors (Hungary) [5]. Different types of financial incentive packages including bursary projects requiring a length of service equivalent to the length of the bursary are popular financial-type retention measures [17]. The impact of these is hardly measurable on a short term, but signs of improved retention can be experienced [22]. Obviously, there is a constant need to analyse drivers of international recruitment within the major destination countries. In addition, debates and dialogues need to be stimulated on how the implementation of the Code can be in the interest of both destination and source countries [23] and all stakeholders within the EU.

Concerning the need to improve the use of EU financial mechanisms for health workforce retention to support ethical solutions, a mapping study on the use of EU structural and investment funds already shows some positive tendencies [24]. Examination of current and previous programming period shows that health is a relevant issue both for European Regional Development Fund and European Social Fund (though having no health operational programme). Investments prevail in the “new” Member States, with a shift in focus from investment in health infrastructure to community-based care, access to care and active and healthy ageing. Health workforce (including inter alia training, lifelong learning, workforce planning, retention) is even explicitly proposed recently as thematic block of a DG SANTE tender from the Health Programme (published in December 2015) [25]. However, while making better use of available resources, the possibility to establish a European fund to compensate training costs in source countries is still an idea [7, 26] to consider.

In order to better manage mobility, bilateral solutions should be established as it would be advantageous for the receiving country and the migrants themselves. Institutional level cooperation like the Hungarian-Swedish example can offer the possibility to include ethical principles such as not offering long-term posts for staff without the consent of the other party. Such small-scale successful experimental programme can be further extended via a framework or model programme to more institutions or more professional areas. Even if not much evidence is available, these types of programmes offer the chance to provide for a better distribution of EU workforce by covering short-term shortages as well as a possibility for career development and financial advantages for the individual. However, additional evidence is needed whether such program in the long run facilitates return or rather results in permanent migration and thus creates even larger levels of imbalances. This could possibly be an area where an EU level mechanism could be developed for knowledge and skill transfers between Member States [7, 26].

International data collections facilitate the availability of reliable information on inward migration when collecting information from receiving countries on their foreign workforce, as the OECD/WHO/Eurostat Joint Questionnaire on non-monetary healthcare statistics introduced. Global examples of stable data-exchange structures work for example in the form of government-level bilateral agreements, as demonstrated by Indonesia, where a surplus of nurses and midwives triggered formal governmental agreements with a few Middle East countries and Japan to recruit their nurses, including the development of information systems on number and profiles of migrated health personnel. [27]. The final recommendation of the Joint Action on mobility data elaborated further the idea of a system of feedback proposing information provision on health professionals who became eligible to practise. The possible methods for this cooperation should be investigated; examples could be online tools and bilateral exchanges tested by pilot projects [21].

### Limitations

Discussions took place on the basis of relevant issues collected by participants, where subjective decisions might have taken place. Evaluation of the feasibility and relevance of the discussed topics categorising them as having “top”, “high”, “medium” and “low” importance was arbitrary since it gave high and medium importance for issues having only a couple of votes (above 10 and 5 respectively). However, this approach was justified by the fact that all issues originate out of a selection in an earlier phase and were as such considered as relevant. Some people did not even use “0” mark to demonstrate the importance of all questions raised.

## Conclusions

Although the WHO Code itself and its implementation is not aimed to address growing inequalities and deepening HWF imbalances within the EU, its principles are also relevant within a free movement zone. The contribution of the Joint Action to knowledge-sharing and building an arena for discussion between various types of stakeholders was of very high value. The following areas have been proposed to first concentrate on within the EU when applying the Code: tailor-made bilateral cooperations including solutions of circular mobility and cross-border training; better use of EU financial resources; improved retention and integration; and better data and monitoring to provide evidence which can substantiate decision-making.

Implementation practices of the Member States show positive effects of adhering to the provisions of the Code and also provide useful ideas to intra-EU mobility management which are compatible with EU law or could be adjusted to it. Systematic steps of planning should thus be reconciled with global requirements reflected in the WHO Code and the WHO global health workforce strategy adopted in May 2016 [28].

Retention policies were categorised as top priority within the free movement context. As even considerable pay rise cannot compete with salaries offered in countries with considerably higher remuneration levels, not only source countries should offer a complex package of incentives, but also incentivising destination countries to train and retain their workforce is essential. Commitment has to be strengthened to apply and adapt global experiences for intra-EU situations. To support this, long-term benefits of respecting global principles should be better demonstrated, and more evidence on intra-EU solutions is needed. A debate on how to incentivise all parties to follow such long-term objectives should be facilitated at the EU level. Existing structures are good starting points for information exchange at the EU level. Establishing however stable bilateral structures can also be considered.

The unfinished agenda of the applicability of the WHO Code for EU is undoubtedly a major topic for future deliberations.
